# Integration of pharmacometabolomics with pharmacokinetics and pharmacodynamics: towards personalized drug therapy

**DOI:** 10.1007/s11306-016-1143-1

**Published:** 2016-12-19

**Authors:** Vasudev Kantae, Elke H. J. Krekels, Michiel J. Van Esdonk, Peter Lindenburg, Amy C. Harms, Catherijne A. J. Knibbe, Piet H. Van der Graaf, Thomas Hankemeier

**Affiliations:** 1 Division of Analytical Biosciences, Systems Pharmacology Cluster, Leiden Academic Centre for Drug Research, Leiden University, Einsteinweg 55, 2333 CC Leiden, The Netherlands; 2 Division of Pharmacology, Systems Pharmacology Cluster, Leiden Academic Centre for Drug Research, Leiden University, Leiden, The Netherlands; 3 Certara QSP, Canterbury Innovation Centre, Canterbury, UK

**Keywords:** Personalized medicine, Pharmacology, Pharmacokinetics, Pharmacodynamics, Pharmacometabolomics, Metabolomics, Biomarker

## Abstract

Personalized medicine, in modern drug therapy, aims at a tailored drug treatment accounting for inter-individual variations in drug pharmacology to treat individuals effectively and safely. The inter-individual variability in drug response upon drug administration is caused by the interplay between drug pharmacology and the patients’ (patho)physiological status. Individual variations in (patho)physiological status may result from genetic polymorphisms, environmental factors (including current/past treatments), demographic characteristics, and disease related factors. Identification and quantification of predictors of inter-individual variability in drug pharmacology is necessary to achieve personalized medicine. Here, we highlight the potential of pharmacometabolomics in prospectively informing on the inter-individual differences in drug pharmacology, including both pharmacokinetic (PK) and pharmacodynamic (PD) processes, and thereby guiding drug selection and drug dosing. This review focusses on the pharmacometabolomics studies that have additional value on top of the conventional covariates in predicting drug PK. Additionally, employing pharmacometabolomics to predict drug PD is highlighted, and we suggest not only considering the endogenous metabolites as static variables but to include also drug dose and temporal changes in drug concentration in these studies. Although there are many endogenous metabolite biomarkers identified to predict PK and more often to predict PD, validation of these biomarkers in terms of specificity, sensitivity, reproducibility and clinical relevance is highly important. Furthermore, the application of these identified biomarkers in routine clinical practice deserves notable attention to truly personalize drug treatment in the near future.

## Introduction

One of the main challenges in modern drug therapy is that a single compound with one fixed dose does not optimally treat all individuals in a population that suffer from a specific disease in a population. Knowledge about inter-individual differences in drug pharmacology and descriptors of these differences is essential to treat all individuals in a population effectively and safely. Individual variations in the response to drug treatment may result from genetic polymorphisms (Weinshilboum 2003) or other epigenetic factors, environmental factors including diet, life style, earlier/current drug treatments, and microbiome (Li and Jia 2013). Furthermore, demographic characteristics like age, sex, and bodyweight, or disease related factors like disease status and treatment-related factors (Alomar 2014) may cause variation. It is not known a priori which of these factors will best predict the clinical outcome of a treatment in an individual.

Personalized medicine, also sometimes referred to as precision medicine, aims to offer a tailored drug treatment to achieve the most optimal therapeutic effects with the least amount of adverse effects for each individual (Schork 2015). Information provided by predictors of expected individual responses to a particular drug, can inform clinicians in the decision making process for drug selection and drug dosing regimen (Kitsios and Kent 2012). And we are convinced that pharmacometabolomics will allow to find metabolic predictors for drug selection and drug dosing as will be elaborated in this paper.

## Interplay between drug pharmacology and patients’ (patho)physiology

**Fig. 1:**
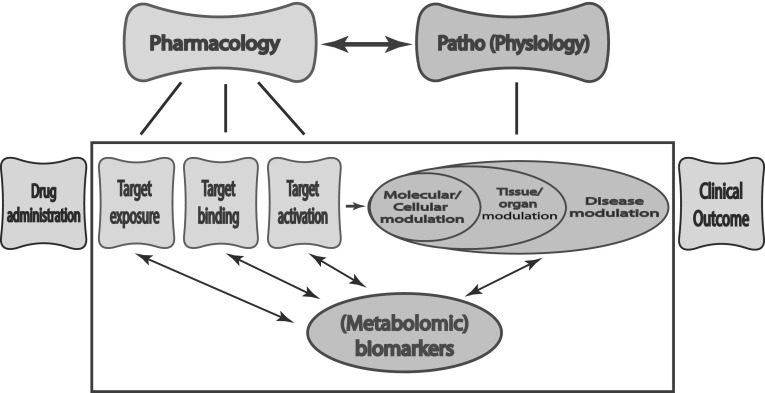
Potential of metabolomics in the interplay between drug pharmacology and patients (patho)physiology

For optimal pharmacotherapy, the interplay between drug pharmacology and patients’ (patho)physiology needs to be understood (van der Greef and McBurney 2005; Vicini and van der Graaf 2013). As illustrated in Fig. 1, important aspects regarding drug pharmacology are target exposure, target binding, and target activation, these processes are governed by drug-specific properties and patient characteristics. Modulating (patho)physiological biochemistry networks on a cellular level and, more importantly, on a tissue or organ level, or systemic level, will eventually determine the treatment outcome for a patient. The target activation triggers modulation of the (patho)physiological system including self-regulatory feedback mechanisms of the patient, also called downstream effects, and these modulations can vary between patients. When studying the effects of pharmacotherapy, none of the processes in the chain from drug administration to patient outcome (Fig. 1) should be studied in isolation. Rather, all aspects need to be considered in the context of their causal and temporal interactions. Moreover, in order to achieve truly personalized medicine, inter-individual variability throughout these processes need to be quantified and predictors for inter-individual difference identified.

Biomarkers are important tools to study the complex interplay between drug pharmacology and the patients’ (patho)physiology, both on a population level and on an individual level. In this context, biomarkers are “a measure that characterizes, in a strictly quantitative manner, a process, which is on the causal path between drug administration and effect” (Danhof et al. 2005). A variety of biomarkers currently exist and endogenous metabolites, or more probably metabolite fingerprints (Adourian et al. 2008; van der Greef and McBurney 2005), are promising for providing novel biomarkers for all processes on the causal chain between drug dose and patient outcome as outlined in Fig. 1.

If a drug does not reach its target, it cannot elicit its effect. Thus, drug concentration in blood is a biomarker for target exposure. Because drug concentrations at the target site may be difficult to measure, drug concentration in plasma is often used as a convenient surrogate measure for target exposure. However, in some cases where a compound has a peripheral target site (e.g. in brain), drug concentrations in blood cannot fully predict the target exposure.

Pharmacokinetics (PK) is the study of drug absorption, distribution, metabolism and excretion. Pharmacokinetic profiles describe the time course of drug concentrations in plasma or other tissues or biological matrices. Total drug exposure in a certain tissue is represented by the area under the concentration curve (AUC). The processes underlying drug PK are quantified using primary parameters like clearance (CL) and distribution volume (V), or secondary parameters like absorption or distribution rate constants. Inter-individual variability in biological processes results in variability in the drug PK profiles between individuals. The ability to predict individual variations in the PK of drugs prior to drug administration is of interest in order to avoid over-dosing (e.g. adverse effects) and under-dosing (e.g. therapeutic failure). Currently, demographic, disease-related or treatment-related factors including for instance age, bodyweight, disease state, drug formulation, and concomitant drug therapy, are used as predictors of quantitative inter-individual differences in the PK of drugs. These predictive factors of variability are called covariates.

Current methodologies using conventional covariates alone can often explain a large part of the inter-individual variability in the PK of a drug (Joerger 2012). However, this may not be sufficient for drugs with a narrow therapeutic window and relatively high unexplained inter-individual variability. In these cases, therapeutic drug monitoring (TDM) provides a viable option to improve an individuals’ pharmacotherapy. With TDM, dose adjustments are made based on additional information on the individual PK parameter values, derived from relevant exposure measures after one or multiple drug doses have been administered.

There are, however, situations where the use of conventional covariates, even in combination with TDM, is not sufficient to guide drug dosing for target exposure attainment. In critically-ill patients, patients with organ failure, or in the end-of-life stages of terminal patients, many (patho)physiological changes and drug–drug interactions occur in quick succession, and all or a number of these factors may influence the PK of administered drugs. In these vulnerable patients, the exact clinical status is difficult to assess using conventional measures, but this information may be essential to predict the individual PK parameter values needed to provide personalized drug dosing.

To elicit an effect, a drug should not just reach a target, it should also bind to the target and activate the target to initiate the desired biosignal that will for instance lead to changes in enzyme activity or changes a (patho)physiological pathway. Factors that influence these interactions include both the physicochemical properties of the drug and the phenotype of the patient. Inter-individual differences in the target phenotype may influence target binding and activation and thereby the eventual treatment outcome in individual patients.

The biosignal initiated by target activation is subsequently potentiated into the (patho)physiological system of the patient. Potentiation of this signal may involve relatively simple molecular pathways, but often involves complex interacting networks related both to healthy physiology and disease-related pathophysiology, that will yield modulations on cellular, tissue and organ levels, and on the disease state level. A wide range of these factors related to (epi)genetic factors and patient phenotype may result in inter-individual variability in signal potentiation and thereby patient outcome.

Drug pharmacodynamics (PD) is related to the effect of drugs and in PK-PD analysis the relationship between drug concentration, usually in blood, and treatment outcome is defined. Usually, inter-individual variability in PD processes exceeds the inter-individual variability in PK. Failure to properly account for the variability in the PD of a drug may cause therapy failure or toxicity for individual patients (Levy 1998).

As indicated in Fig. 1, drug effects, either desired or unwanted side-effects, can be described on different levels (from target exposure, to target binding and target activation, to ultimately disease modulation leading to clinical outcome) and the relationships between drug concentration and effect will become less direct and potentially more complex when moving downstream in signal potentiation, and ultimately to clinical outcome. Especially in complex diseases of which the mechanism is not yet fully understood, for instance psychological and neurological diseases, there is a strong need for validated biomarkers to assess disease progression. In addition, such a biomarker can be informative about the impact of pharmacotherapy on various levels of the (patho)physiological system.

Similar to PK, conventional covariates can be used as predictors for inter-individual variability in drug effects, but, similar to TDM, in PD they may not always be sufficient. Moreover, also similar to TDM, dose adjustments and even drug selection should be guided based on prediction of individual patient outcome, particularly for drugs in which the drug effects are difficult to quantify or delayed (e.g. psychoactive drugs, cytostatics, antidepressants, etc.). Another example where we need proper PD prediction is the use of drugs in vulnerable or critical patients. In these cases, validated biomarkers that can predict long-term outcome based on short-term changes are needed to facilitate individual optimization of pharmacotherapy.

As outlined above, optimal pharmacotherapy requires information on the current (patho)physiological status of an individual patient. Conventional covariates may be useful in this respect and may even be sufficient in predicting individual deviations from population responses, but when these don’t suffice, other methods for the establishment of phenotypic profiles may be required. Such methods are preferably prospective and minimally invasive.

One way of PK phenotyping involves the administration of a probe drug or drug cocktail to assess the phenotype of several drug-metabolizing enzymes (Sharma et al. 2004). However, this is not always feasible, especially in vulnerable populations, and this involves prolonged clinical visits and increases time and cost of treatment. An alternative phenotyping approach would be using endogenous biomarkers that could predict enzyme activity without risk, time, and cost of exogenous drug administration. Another advantage of endogenous biomarkers is that retrospective analysis of banked samples can be conducted. Identified predictive endogenous biomarkers can be used as covariates to guide clinicians in decision making regarding treatment options by using minimal amounts of biological fluids. In this regard, the application of metabolomics can serve as an alternative or additional method to the current clinical practices to achieve personalized treatment (Bernini et al. 2009; Fernie et al. 2004; Guo et al. 2015; Schnackenberg 2007; Suhre et al. 2011; van der Greef et al. 2006).

Metabolomics or endogenous metabolite profiling may be used as a phenotypic tool to provide accurate information on the current (patho)physiological status of patients to prospectively inform on individual differences in both PK and PD processes and thereby guide drug selection and drug dosing. Furthermore, in cases where the exact mechanism of a disease or drug effect is unknown, endogenous metabolites and their change after administration of a drug can provide mechanistic insight in disease status and drug response of an individual (Kaddurah-Daouk et al. 2008).

## Metabolomics and pharmacometabolomics

Pharmacogenomics (PG), which uses genetic polymorphisms to predict individual variations in responses to drugs, for instance to classify patients as poor or rapid drug metabolizers, or drug responders or non-responders (Evans and McLeod 2003; Evans and Relling 2004; Pirmohamed 2014) has been increasingly used to inform personalized medicine. However, studying a patient’s genotype does not always allow for a clear definition of a phenotype, nor does it give information about the current (patho)physiological state of an individual (Carr et al. 2014), as the genotype does not capture time-varying processes influenced by environmental factors and/or disease-related factors.

Metabolomics offers an advantage over PG in explaining the inter-individual variability in drug PK or PD, as it provides a direct readout of the current metabolic state of an individual. The endogenous metabolite profile is a snapshot of the phenotypic status of an individual resulting from, for instance, demographic factors, environmental interactions, microbiota, or disease status. Pharmacometabolomics is emerging as a discipline of metabolomics that studies the interplay between drug pharmacology and the patients’ (patho)physiology, by measuring endogenous metabolites that inform on variability in the drug PK or PD phenotype (Clayton et al. 2006; Kaddurah-Daouk and Weinshilboum 2015; Lindon et al. 2006; Nicholson et al. 2011). This concept has first been illustrated in rats, in a study that showed that metabolomic information in pre-dose urine samples is predictive of both drug metabolism (PK) and toxicity (PD) of paracetamol (Clayton et al. 2006).

So far, pharmacometabolomic research addresses:

1. The identification of endogenous metabolites for predicting individual drug PK characteristics (Huang et al. 2015; Kienana et al. 2016; Phapale et al. 2010; Rahmioglu et al. 2011; Shin et al. 2013; Tay-Sontheimer et al. 2014).
2. The identification of endogenous metabolites and their metabolic pathways for predicting individual drug PD characteristics (Condray et al. 2011; Kaddurah-Daouk et al. 2010, 2011a, b; Kaddurah-Daouk and Weinshilboum 2014; Keun et al. 2009; Krauss et al. 2013; Trupp et al. 2012; Yerges-Armstrong et al. 2013; Zhu et al. 2013).
3. The identification of endogenous metabolite biomarkers for monitoring disease progression and pharmacotherapy in individual patients (Backshall et al. 2011; Kinross et al. 2011; Nicholson et al. 2012).

The analytical methods applied to discover metabolic biomarkers use advanced and sensitive analytical instruments such as NMR, LC-MS and GC-MS (Chen et al. 2007; Dona et al. 2014; Emwas et al. 2013; Garcia and Barbas 2011). Metabolomics research can be conducted either in a targeted or un-targeted fashion. In targeted approaches, pre-selected endogenous metabolites, which belong either to defined chemical classes or to particular metabolic pathways, are quantified. In an untargeted approach or a global profiling approach, endogenous metabolites are quantified in an unbiased fashion without any pre-selection of metabolites. This mode is advantageous in exploratory studies, which can generate hypotheses as well as generate data for biomarker discovery. However, in an untargeted metabolomic analysis, the physicochemical properties of the metabolites determine whether they can be in principle extracted and detected, and quantitative information on endogenous metabolite concentration is often less precise than in targeted analysis, and comparability between studies and labs is less straightforward than in targeted analysis, where absolute concentrations are reported for targeted metabolites for which a calibration model is available.

For the discovery of metabolite biomarkers to identify predictors of PK and/or PD variability or for monitoring the disease progression and the modulation of the disease progression by pharmacotherapy, disease metabolic phenotypes or pre- and post-dose metabolic phenotypes are often established. These metabolic phenotypes offer unique read-outs that contain information about the (patho)physiological state of an individual at particular time points. The optimal design of the metabolomic biomarker discovery study, will depend on the intended use of the biomarker, which could include for instance identification of responders and non-responders to drug treatment, predict individual PK variability or assess drug–drug interactions. The statistical models of the metabolomics data will then integrate both causal and temporal information. Ultimately, the metabolite biomarkers may be used in routine clinical practice, either prospectively to guide drug selection and drug dose selection based on pre-dose metabolite biomarker profiles, or retrospectively to monitor the effects of pharmacotherapy.

**Fig. 2:**
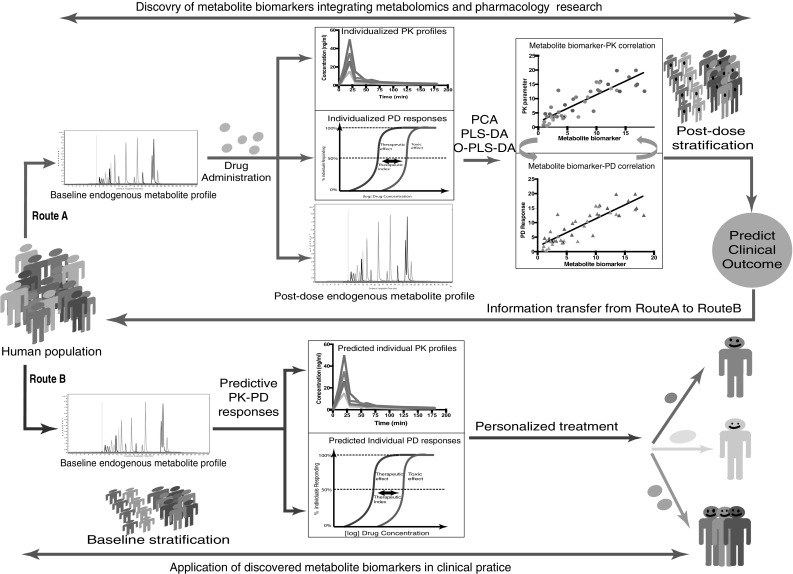
Pharmacometabolomics in research (*route A*) and clinical practice (*route B*). *Route A* (*red*) discovery of metabolite biomarkers to predict pharmacological treatment outcome using statistical methods that couple data from metabolomics profiling to PK and / or PD variables of an individual. *Route B* (*blue*) prospective application of metabolite biomarkers in routine clinical practice, using information from *route A* for personalized treatment

Figure 2 illustrates the use of pharmacometabolomics in both research (route A) and clinical practice (route B). Route A illustrates how pharmacometabolomics can be coupled to the clinical outcome variables of pharmacotherapy to investigate and identify metabolomic biomarkers for drug pharmacology and the interaction with patients’ (patho)physiology. In this route, pre-dose and post-dose endogenous metabolite profiles are obtained together with individual PK and PD variables. Using a range of multivariate statistical methods such as for instance principal component analysis (PCA), partial least squares discriminant analysis (PLS-DA) and orthogonal partial least squares discriminant analysis (O-PLS-DA), correlations between endogenous metabolites and pharmacological characteristics can be investigated (Bartel et al. 2013). Further research is then focused on identifying the role of the biomarker in the causal chain between drug administration and patient outcome, for instance using network analysis (Kotze et al. 2013) and/or population models that quantify the time course of drug concentration and drug effect (Gabrielsson et al. 2011; Wright et al. 2011). Route B illustrates how the pharmacometabolomic information obtained in route A is prospectively applied in clinical practice to personalize drug treatment. Using pre-dose samples and the quantitative knowledge on the relationships between endogenous metabolites and pharmacological outcome, pharmacotherapy, in terms of drug selection and dose selection, can be tailored to an individual.

## Pharmacometabolomics informs pharmacokinetics

Target exposure is one of the first steps on the causal chain linking drug dosing to patient outcome. Knowledge on the sources and extent of inter-individual variability and the availability of descriptors for variability, will allow clinicians to prospectively adjust drug doses for individual patients.

The main aim of pharmacometabolomic studies related to drug PK is to identify endogenous metabolite markers that allow for the stratification of patients into exposure groups, which is needed to individualize drug dosing regimens. Factors that are known to have marked impact on the PK of drugs include, for instance, expression and activity of drug metabolizing enzymes, tissue composition including the expression of drug binding plasma proteins and tissue proteins, drug transporters, and gut microbiome.

One of the first reported human studies linking pre-dose metabolomics information in urine to drug exposure measures was performed in healthy volunteers taking tacrolimus (Phapale et al. 2010). Tacrolimus is an immunosuppressant used during organ transplantation and has a narrow therapeutic index with a high degree of inter-individual variability in its PK. As dose adjustments of this drug are futile by the time over-dosing (e.g. organ toxicity) or under-dosing (e.g. organ rejection) become apparent, accurate exposure monitoring or prediction is important. In the study, the authors used first untargeted metabolomic profiling and multivariate statistics to correlate endogenous urine metabolites to the AUC of tacrolimus. Then a hypothetical molecular network was developed that included the obtained metabolic biomarkers, and findings on important modules in this network were linked to mechanistic knowledge of the underlying PK processes for tacrolimus to select possibly causal biomarkers. From this, a metabolomic phenotype based on pre-dose urine concentrations of four endogenous metabolites was derived that can predict a patients’ exposure to tacrolimus, thereby allowing a prospective individual dose selection.

Another report linked pre-dose plasma metabolomic profiles to exposure measures of atorvastatin in healthy volunteers (Huang et al. 2015). Atorvastatin is an HMG-CoA reductase inhibitor for which considerable inter-individual variability in drug metabolism leads to up to 45-fold differences in plasma concentrations leading to therapy failure in some and adverse effects in others. In this study, the authors first applied untargeted profiling of metabolites with GC-MS and PLS analysis to establish a model that predicts endogenous metabolites and pharmacokinetic parameters (C_max_ and AUC). Using selected metabolites, hypothetical metabolic networks were constructed to visualize the role of metabolite pathways explaining the variability. Later, an O-PLS model was used to stratify the individuals into subgroups based on the pre-dose metabolite behavior. For atorvastatin conventional covariates have proven to be suboptimal in predicting individual exposure measures and this study showed a combination of endogenous metabolite biomarkers to have increased predictive value for this drug.

As drug metabolizing enzyme activity is an important contributor to drug clearance, and as drug clearance is a major determinant for exposure, some (pharmaco)metabolomic studies investigate endogenous metabolomic predictors for drug metabolizing enzyme activity in general. It is, however, important to note that other factors, including hepatic blood flow, plasma protein binding, and hepatic transporters, also influence drug metabolic clearance. These factors may limit the direct translation of findings regarding drug metabolism of one probe compound to other compounds that are substrates for the same enzymes.

When focusing on the metabolism, CYP3A enzymes are responsible for the metabolism of the majority of prescribed drugs. These enzymes have multiple functional alleles and they are subject to induction and inhibition by various exogenous compounds. The interaction processes are highly variable between individuals and become especially relevant in patients taking multiple drugs. To investigate the applicability of pharmacometabolomics in prospectively informing on induction of CYP3A4 metabolism, Rahmioglu et al. performed a study correlating pre-dose metabolomic urine measures to quinidine metabolite ratios after CYP3A4 induction with Hypericum perforatum, known as St. John’s Wort (Rahmioglu et al. 2011). Endogenous urinary metabolite measures were identified that were predictive of the quinidine metabolite ratio [3-hydroxyquinine to quinine (3OH-Q:Q)], but they all remained empirical predictors as none of these could be mechanistically linked to CYP3A4 activity. A potential explanation for this is that ratios in drug metabolite concentrations are dependent on both formation and elimination rates of metabolites, making this measure not very specific for enzyme activity alone.

A more recent study in healthy male volunteers used a more direct measure for CYP3A activity by investigating the clearance of midazolam, a drug that is known to be predominantly cleared through CYP3A-mediated metabolism. Moreover, this study not only investigated scenarios after CYP3A induction using ketoconazole, but also included situations without drug interactions or with inhibition using rifampicin (Shin et al. 2013). The authors were able to identify an endogenous steroid metabolomic profile in urine which could accurately predict midazolam clearance under all investigated conditions. A link between steroid metabolism and CYP3A activity had already been established, but this study defined a more predictive biomarker profile. Moreover, the authors showed that timing of urine collection in each treatment phase did not influence the predictive value of the biomarker, suggesting that it can be reliably used to establish current midazolam clearance in patients that are already receiving drug therapy.

In the paediatric population, on top of genetic, environmental and disease-related factors that are also present in adults, growth and development results in continuous changes in physiological processes underlying drug PK and PD. Much research efforts have focused on quantifying the influence of these changes on the PK, and to a lesser extent PD, of drugs in children. A recent study by Tay-Sontheimer et al. (Tay-Sontheimer et al. 2014), illustrated a first attempt to use pharmacometabolomic approaches to prospectively and non-invasively predict drug clearance of a CYP2D6 substrate in this population as well. Strong conclusions cannot yet be made based on this study, since parent and drug metabolite ratios were used to define enzyme activity. Other limitation in this study were encountered as the identified endogenous metabolite biomarker Ml, could not be structurally identified based on its fragmentation spectra, and most importantly the concentration of the endogenous metabolite biomarker was below the detection limits in samples of poor metabolizers. However, the idea of using pre-dose pharmacometabolomic measures to prospectively determine drug doses in the pediatric population is appealing.

As most drug metabolism occurs in the hepatocytes of the liver, influx and efflux transporters in these cells may influence the metabolic clearance of their substrates. Moreover, efflux transporters in hepatocytes may offer an alternative clearance route by transporting drugs directly into the bile. Also within nephrons of the kidneys, active transporters may facilitate drug excretion or reuptake. Finally, intestinal drug uptake and tissue distribution of drugs may be influenced by transporters. As with enzymes, genetic polymorphisms in drug transporters may influence their activity (Kerb 2006) and interactions with endogenous or exogenous compounds may induce or inhibit the transporters in a time-dependent manner (Konig et al. 2013).

In a recent study using an untargeted metabolomics approach, a pre-dose urinary metabolomic profile based on 28 endogenous metabolites was identified that was predictive of the clearance of high-dose methotrexate in patients with lymphoid malignancies (Kienana et al. 2016). Inter-individual and inter-occasion variability in the clearance of methotrexate is large, leading to regular toxicity events in patients. Many of the 28 identified endogenous metabolites are substrates for organic anion transporters in the kidney, transporters that are also known to play a major role in the elimination of methotrexate, suggesting that metabolomic profiles may also provide information on the function of transporters at a given time-point.

Recently it has been recognized that the human gut microbiome may contribute to variations in the response to drug treatment, for instance by the bacterial synthesis of unique metabolites from administered drugs or their metabolites. In a study of paracetamol, Clayton et al., demonstrated that formation of *p*-cresol by the gut microbiome results in a competitive interaction for the systemic sulphation of paracetamol, causing a decreased relative sulphation of the drug (Clayton et al. 2009). Given that the therapeutic window of paracetamol is wide, this finding may not be of big relevance for this specific drug, but it may have important implications for other drugs that have a narrow therapeutic window and large inter-individual differences in drug metabolism. Also for simvastatin, a relationship between pre-dose levels of secondary bile acids produced in the gut and drug effect has been identified (Kaddurah-Daouk et al. 2011a, b), although the exact mechanism underlying this finding is not yet known. The identified secondary bile acids correlated with the concentration of simvastatin, and interactions of these bile acids with (patho)physiological mechanisms are also proposed to influence patients’ responses to simvastatin treatment. As the microbiome of individuals may vary over time, the results of these studies suggest that pharmacometabolomics may provide relevant information on the status of the microbiome of a patient at a given time and the expected effect this has on pharmacotherapy.

## Pharmacometabolomics informs pharmacodynamics

The majority of pharmacometabolomic studies are focused on drug PD and changes in (patho)physiology upon drug exposure. The potential of this type of pharmacometabolomic research has been highlighted for instance in neuropsychiatric diseases (Kaddurah-Daouk et al. 2007, 2011a, b, 2013; Quinones and Kaddurah-Daouk 2009; Yao et al. 2010; Zhu et al. 2013), neurodegenerative disorders (Kaddurah-Daouk et al. 2013), cardiovascular diseases (Kaddurah-Daouk et al. 2010, 2011a, b; Krauss et al. 2013; Trupp et al. 2012), cancer (Backshall et al. 2011; Dang et al. 2009; Keun et al. 2009), chronic kidney disease (Zhao et al. 2014) and hematology (Ellero-Simatos et al. 2014; Yerges-Armstrong et al. 2013).

Most pharmacometabolomic studies in PD set out to investigate the effects of pharmacotherapy by investigating differences in pre and post-dose endogenous metabolomic profiles and identifying patterns that can explain inter-individual differences in treatment outcome. However, as is illustrated in Fig. 1, changes in various levels of the (patho)physiological system induced by pharmacotherapy should not be regarded as static or be studied in isolation, as they only form one link in the context of their causal and temporal interaction in all the processes between drug administration and patient outcome. The studies establishing a link between a specific endogenous metabolite or metabolite profile and treatment outcome of pharmacotherapy should therefore be followed up by extensive studies into the behavior of the new metabolite biomarker under various pharmacological interventions of the (patho)physiological system to validate the specificity, sensitivity, reproducibility, and clinical relevance of the new metabolite biomarker. These validation studies should focus on the influence of different drug doses or drug concentrations on the newly identified metabolite biomarker to establish a concentration-effect relationship, on understanding the causal and temporal relationships of changes in the new metabolite biomarker and other biomarkers or measurements of treatment outcome, and on the response of the new metabolite biomarker to pharmacotherapy with both agonist and antagonist agents. Population modeling approaches can provide a useful tool in quantitatively integrating all the information obtained in the various investigations.

## Pharmacometabolomics informs the clinician

Although metabolomics studies have identified a number of metabolite biomarkers that can describe and predict inter-individual variability in the PK or PD of drugs, the application of the obtained knowledge in clinical practice remain relatively limited. Validation of promising metabolite biomarkers is therefore urgently required to prove their specificity, sensitivity, reproducibility and clinical relevance. Once the understanding of causal and temporal relationships and inter-individual variability is there, the obtained knowledge needs to be taken to the clinic to optimize drug therapy.

It is worth to mention that pharmacogenomics studies have played an important role in predicting drug PK in the last decade, but pharmacogenomics does not provide information about the current (patho)physiological state of an individual, and take environmental factors into account. As one’s individual genome will be soon available for many persons, pharmacogenomics is very attractive for PK prediction. However, there are many cases where pharmacogenomics was not able to predict PK, and where pharmacometabolomics is an attractive alternative. The reason is that pharmacometabolomics provides a snapshot of the phenotypic status of an individual resulting from, for instance, demographic factors, environmental interactions, microbiota, or disease(s) status. We anticipate that pharmacometabolomics and pharmacogenomics are very complementary techniques, which we expect will be often combined ultimately in clinical decision support for PK (and PD) prediction.

For the implementation of metabolic biomarkers in the clinical lab there are different requirements: (1) metabolites should be reported as absolute concentrations, (2) the analysis should be cost-effective and (3) results should be available to the clinicians in a timely manner. Implementation can occur in two different ways, via targeted analysis, specific cost-efficient assays covering only a limited number of required metabolites, or a broader panel allowing for a general metabolomics assay covering hundred or more (identified) metabolites that inform about the general health state including the prediction of treatment outcome. For the targeted assay, we can expect that small analyzers will be developed. This might be based on aptamers, miniaturized NMR or mass spectrometers, and might be even handheld. For the broader clinical metabolic profile most probably a lab-based metabolite analyzer using a cost-efficient mass spectrometer will be used. The more metabolite biomarkers will become validated, the more attractive it will be to implement metabolite profiling in the clinical lab for clinical decision support, and we are convinced that with the significant increase of metabolomics studies reporting metabolic biomarkers for PK/PD this will become routine over some years.

It is worth mentioning that pharmacometabolomics can also inform the drug researcher on variation of PK or PD in early clinical studies, especially whether (1) a drug is exposed to the target, (2) whether the drug engages with the target and (3) whether the drug modulates the target in the desired manner. However, this aspects were not the subject of this review.

## Conclusion and future recommendations

An interplay between many factors related both to drug pharmacology and a patients’ (patho)physiology is responsible for the drug treatment outcome for the patient. Variability in all these processes can yield variability in treatment outcome. While conventional covariates are often sufficient in prospectively informing treating physicians on the PK of individuals, pharmacometabolomics has proven to have additional value in prospectively informing PK and aiding in prospective drug dose individualization when conventional covariates cannot explain inter-individual variability sufficiently. Compared to pharmacogenomics, pharmacometabolomics takes the actual health state into account. This is especially important in critically-ill patients, the elderly or terminal patients where many drug–drug interaction occurs, and also for drugs with a narrow therapeutic window and relatively high unexplained inter-individual variability, and where you want to predict outcome before starting any treatment rather than using therapeutic drug monitoring to modify the treatment regime. This may be particularly relevant in patient populations taking multiple drugs or which rapidly change their health state, such as patients with organ failure or organ transplants, but also in pediatric or elderly patients or pregnant women.

Pharmacometabolomics can aid in predicting pharmacodynamics and ultimately clinical treatment outcome. As (patho)physiology changes during disease and pharmacotherapy, it is important to not consider the endogenous metabolites as static variables in isolation. The influence of drug dose and temporal changes in drug concentrations and metabolite network interactions should be part of these investigations as well, before endogenous metabolite markers can be considered for informing drug selection or drug dose selection in patients. Therefore, both PK and PD, should be included in pharmacometabolomics studies, where currently often PK is not considered in many PD pharmacometabolomis studies.

In conclusion, pharmacometabolomics is very promising for predicting pharmacokinetics and pharmacodynamics, and if we can manage to incorporate findings in this field in clinical practice, we are able to realize personalized medici.
